# Long-term low dose dietary resveratrol supplement reduces cardiovascular structural and functional deterioration in chronic heart failure in rats^1^

**DOI:** 10.1139/cjpp-2016-0512

**Published:** 2016-12-17

**Authors:** Ismayil Ahmet, Hyun-Jin Tae, Edward G. Lakatta, Mark Talan

**Affiliations:** Laboratory of Cardiovascular Sciences, Intramural Research Program, NIA, NIH, Baltimore, MD 21224, USA.

**Keywords:** heart failure, resveratrol, echocardiography, remodeling, pulse wave velocity

## Abstract

A short-term exposure to resveratrol at high dosages exerts a remarkable cardioprotective effect. Whether a long-term exposure to resveratrol at low dosages that can be obtained through consumption of a resveratrol-rich diet is beneficial to heart diseases is unknown. We tested the effects of a resveratrol-enriched diet on cardiovascular remodeling of chronic heart failure (CHF) in rats resulting from permanent ligation of left coronary artery. Two weeks after surgery, rats were started on either a resveratrol-enriched (R; 5 mg/kg per day; *n* = 23) or normal (Control; *n* = 23) diet for next 10 months. Serial echocardiography in Control showed a significant decline in LV ejection fraction, increases in LV end-systolic and end-diastolic volumes, and expansion in myocardial infarct from pre-treatment values. In R, compared with Control, there were substantial improvements in those parameters. End-point LV pressure-volume loop analysis showed a significantly improved LV systolic function and AV-coupling, an index of energy transfer efficacy between the heart and aortic tree, in R compared with Control (*p* < 0.05). Aortic pulse wave velocity, a measure of arterial stiffness, was significantly lower in R (389 ± 15 cm/s; *p* < 0.05) compared with Control (489 ± 38 cm/s). These results demonstrated that long-term dietary resveratrol supplement reduces cardiovascular structural and functional deterioration in CHF.

## Résumé

Une exposition à court terme à des doses élevées de resvératrol exerce un effet cardioprotecteur manifeste. Par contre, on ne sait pas si une exposition à long terme à de faibles doses de resvératrol, comme celles qui peuvent être obtenues avec l’administration d’un régime alimentaire riche en resvératrol, permet d’obtenir des avantages sur le plan des maladies cardiaques. Nous avons étudié les effets d’un régime alimentaire enrichi en resvératrol sur le remodelage cardiovasculaire dans l’insuffisance cardiaque chronique (ICC) provoquée par une ligature permanente de l’artère coronaire gauche chez le rat. Deux semaines après l’intervention chirurgicale, nous avons commencé à administrer aux rats un régime alimentaire enrichi en resvératrol (R; 5 mg/kg par jour; *n* = 23) ou normal (Témoin; *n* = 23) pour les 10 mois suivants. Dans le groupe Témoin, les échocardiographies sériées ont montré la présence d’une nette diminution de la fraction d’éjection du VG, une augmentation des volumes télésystolique et télédiastolique du VG ainsi qu’un élargissement de l’infarctus du myocarde par rapport aux valeurs mesurées avant l’administration du produit. Nous avons observé des améliorations plus substantielles sur le plan de ces paramètres dans le groupe R que dans le groupe Témoin. Ultimement, l’analyse de la boucle pression-volume du VG a montré des améliorations nettement plus importantes sur le plan de la fonction systolique du VG ainsi que du couplage AV — un indice de l’efficacité du transfert d’énergie entre le cœur et l’arbre aortique — dans le groupe R que dans le groupe Témoin (*p* < 0,05). La vitesse des ondes pulsatiles dans l’aorte — une mesure de la rigidité des artères — était nettement moins élevée dans le groupe R (389 ± 15 cm/s) que dans le groupe Témoin (489 ± 38 cm/s; *p* < 0,05). Ces résultats montraient que l’administration à long terme de suppléments alimentaires de resvératrol entraîne une diminution de la détérioration structurelle et fonctionnelle du système cardiovasculaire dans l’ICC. [Traduit par la Rédaction]

## Introduction

Chronic heart failure (CHF) continues to be a major cause of morbidity and mortality (Xu et al. 2010). The prevalence of CHF reaches 20% in older population (Dickstein et al. 2008). In the US, the estimated annual cost of health care attributable to CHF exceeds 35 billion dollars (Stewart et al. 2002; Rosamond et al. 2008). Despite the remarkable progress in CHF treatment over the last 2 decades, the overall annual mortality associated with CHF remains high, at around 10% (Neubauer 2007; Heart Failure Society of America 2006), and the quality of life among survivors becomes dramatically compromised as the disease progresses (Juenger et al. 2002; Hobbs et al. 2002). Thus, a search for novel therapeutic interventions to improve the course of CHF continues.

Beneficial effects of resveratrol, a red wine extract, in many pathological conditions, disease pre-cursors, and aging in different organs and species have been well documented (Guerrero et al. 2009). It also has been shown that resveratrol mimics caloric restriction and exerts similar beneficial effects in aging (Barger et al. 2008; Baur et al. 2006; Lagouge et al. 2006; Mayers et al. 2009). Underlining mechanisms for the protective effect of resveratrol, however, are not fully understood. In the cardiovascular field, remarkable cardioprotective properties of resveratrol against myocardial ischemia–reperfusion injury (Hung et al. 2002; Shen et al. 2006), myocardial infarction (Lin et al. 2008; Robich et al. 2010a), myocardial hypertrophy (Liu et al. 2005; Wojciechowski et al. 2010), cardiac arrhythmias (Chen et al. 2008; Chong et al. 2015), and heart failure (Kanamori et al. 2013; Xuan et al. 2012) have been reported in pre-clinical animal models. These effects, however, were achieved at dosages that range between 10 and 4000 mg/kg resveratrol. The efficacy of resveratrol at lower dosages (<10 mg/kg) is still controversial due to the conflicting reports (Burstein et al. 2007; Chen et al. 2008; Robich et al. 2010b; Wojciechowski et al. 2010). Compared with these high dosages, the dietary dosage of resveratrol via consumption of certain fruit or red wine is merely around 5 mg/kg per day. On the other hand, regardless of dosages, most of the pre-clinical studies were conducted within relatively short-term (<3 months) and exposure times to resveratrol have ranged from minutes to 3 months. Despite the popularity of dietary supplementation with resveratrol (most at dietary dosages) in the health-conscious public, especially among patients with cardiovascular diseases, there is no data on whether long-term (>3 months) consumption of resveratrol at dietary dosages has any beneficial effects in CHF. Thus, the effects of long-term consumption of resveratrol-rich diet on progress of chronic diseases, i.e., cardiac remodeling of CHF, is unknown. We investigated the impact of long-term dietary supplementation with resveratrol at a low dose (5 mg/kg per day) on cardiac structural and functional remodeling in CHF induced by MI in rats.

The post-MI CHF rat model has a close resemblance to CHF in humans. The interactions among oxidative stress, inflammation, hypertrophy, arrhythmia, fibrosis, chamber dilatation, infarct expansion, and functional deterioration that have been characterized in this model (Elsner and Riegger 1995; Gaballa and Goldman 2002; Goldman and Raya 1995) make it an ideal candidate for testing the effects of dietary resveratrol treatment. Accordingly, the objective of this study was to test the effectiveness of 10-month resveratrol-enriched diet in the rat model of post-MI dilated cardiomyopathy.

## Materials and methods

### Experimental design

Male Wistar rats (Charles River Laboratories Inc., Wilmington, Massachusetts, USA), weighing 225–280 g, were housed and studied in conformance with the National Institutes of Health Guide for the Care and Use of Laboratory Animals (8th Edition, 2012), with approval from the Institutional Animal Care and Use Committee. The left descending coronary artery was ligated in 110 rats. An additional 10 rats underwent a sham operation without actual coronary ligation. Two weeks after surgery, myocardial infarct (MI) size was assessed in survivors by echocardiography (Echo). Rats with MI sizes between 20% and −50% were divided into 2 groups with similar MI sizes: the Control group (Control; *n* = 23) received a regular diet; the Resveratrol group (R; *n* = 23) received a resveratrol-enriched regular diet ad libitum. Sham-operated rats (Sham; *n* = 10) received a regular diet. Regular diet was a Standard NIH rat chow (NIH-07; Harlan Teklad, Madison, Wisconsin, USA). Resveratrol (>98%) was purchased from Orchid Pharmaceuticals (Aurangabad, India) and mixed to the Standard NIH rat chow (Dyets Inc., Bethlehem, Pennsylvania, USA). The resveratrol-enriched diet was isocaloric with a regular diet and contained ~0.008%–0.01% of resveratrol. In R group, rats received resveratrol-enriched diet beginning at 2 weeks after coronary ligation, and continued for next 10 months. The target daily dosage for resveratrol in was 5 mg/kg. To maintain this dosage, the daily food consumption was assessed and resveratrol concentration in food was accordingly adjusted every 3 months. Animals were inspected daily for signs of moribundity by a person blinded to dietary assignments. Moribund animals were euthanized, and their hearts were harvested for histological measurements. Daily records of dead or euthanized animals were used to calculate continuous mortality curves. Echo was repeated bi-monthly following the initiation of treatment. At the end of 10-month observation period, invasive hemodynamic measurements were performed following the completion of final Echo. Rats were then euthanized and their hearts and thoracic aortas were harvested for histological evaluation.

### Coronary artery ligation

The surgical procedure was performed as previously described (Ahmet et al. 2004).

### Echocardiography (Echo)

Echo was repeated bi-monthly in all rats following the initiation of treatment. Echo (Sonos 5500, a 12-MHz transducer; Hewlett Packard, Andover, Massachusetts, USA) was conducted under light anesthesia with isofluorane (2% in oxygen) via face mask as described previously (Ahmet et al. 2004). In brief, parasternal long axis views were obtained and recorded to ensure that the mitral and aortic valves and the apex were visualized. Short axis views were recorded at the mid-papillary muscle level. Endocardial area tracings, using the leading edge method, were performed in a 2D-dimensional mode (short and long axis views) from digital images captured on cine loop to calculate end diastolic and end systolic LV areas. End-diastolic volume (EDV) and end-systolic volume (ESV) were calculated by a Modified Simpson’s method. EF was then derived as EF = 100 × (EDV – ESV)/EDV. LV mass (LVM) was calculated from a 2D mode. The MI size at the mid-papillary muscle level was estimated from 2D short axis LV images and expressed as a percentage of the LV endocardial circumference. Infarct area was identified as a sharply demarcated section of the LV free wall that failed to thicken during systole. The length of the akinetic part (MI area) of the LV endocardial circumference was measured from freeze-frame images at end-diastole. Posterior wall thickness was measured from M-mode. All measurements were made by a single observer who was blinded to the identity of the tracings. All measurements were offline averaged over 3 to 5 consecutive cardiac cycles. The reproducibility of measurements was assessed in 2 sets of baseline measurements in 10 randomly selected rats, and the repeated measure variability did not exceed ±5%. Percent changes of ESV, EDV, EF, and MI size from pretreatment baseline value (at 2 weeks after surgery) were calculated and presented for all the time-points.

### Hemodynamic measurements

Invasive hemodynamic measurements were performed at the end of the 10-month observation period. LV pressure-volume loop analyses were conducted as described previously (Ahmet et al. 2005). Rats were anesthetized with isoflurane (2% in oxygen), intubated, and ventilated. Following hemodynamic indices were reported: EF, +*dP/dt*, −*dP/dt*, end-diastolic pressure (EDP), isovolumic relaxation time constant (τ), end-systolic elastance (Ees), preload recruitable stroke work (PRSW), end-diastolic stiffness (Eed), arterial elastance (Ea), and arterio-ventricular coupling (AV coupling).

### Pulse wave velocity (PWV)

PWV was measured at the same time as cardiac LV pressure-volume loop analyses as described previously (Ahmet et al. 2011). Briefly, the left femoral artery was isolated, ligated, and a 1F combined pressure-conductance catheter (Millar Instruments Inc., Houston, Texas, USA) was inserted and advanced to the thoracic aorta (exactly 100 mm from the incision). After recording of several pressure waves and corresponding ECGs, the catheter was withdrawn for exactly 50 mm and data recording was repeated. Using the R wave of the ECG as a time marker, the average time between R waves and starting points of 5 corresponding pressure waves at thoracic and abdominal sections of aorta (exactly 50 mm separated) were measured. The transit time of the pressure wave from upper thoracic aorta to lower abdominal aorta was calculated as the time difference between 2 measurements. Using the distance between 2 points of measurement, the PWV was calculated as 50 mm/transit time.

### Histological acquisition

Histological staining and analyses were performed as described previously (Ahmet et al. 2011). In brief, the hearts were isolated and weighed. Hearts were further cut into 2 pieces through the short axis. The basal half was fast frozen and stored at −80 °C, and the apical half was used for histological analysis. Myocardial tissue segments and aortae were imbedded in the paraffin, sectioned (5 μm), and stained with Masson’s trichrome and hematoxylin & eosin. MI size was expressed as an average percentage of the LV endocardial and epicardial circumferences that were identified as infarction in the Masson’s trichrome stained sections.

Myocyte cell size and density were measured in hematoxylin & eosin stained sections of the LV posterior wall. Only myocytes which nuclei were clearly identified were counted. Myocyte diameter was measured as the shortest distance across the nucleus in transverse cell sections. Diameters of 100 myocytes from 5 randomly selected microscope fields (×200 magnification) from the LV posterior wall were averaged to represent the myocyte diameter of a given specimen. Myocyte density was calculated from the same area in the same fashion.

Myocardial tissue fibrosis was measured in Masson’s trichrome stained sections and was expressed as a fraction of a microscopic field (×100 magnification) of the LV posterior wall. An average of 5 randomly selected fields represented results of a given specimen. Collagen content in the thoracic aortic walls was measured on sections stained with Masson’s trichrome. Digital images of stained sections were obtained from light microscopy and analyzed using a digital imaging analysis system (MCID, InterFocus Imaging Ltd, Cambridge, UK). The collagen content in aortic wall was calculated as a percentage of tunica media area. The person assessing all histological slides was blinded to the source of the slides.

### Statistical analyses

All data are expressed as mean ± SEM. Mortality is reported via Kaplan–Meier survival curves. Differences among survival curves were assessed using Logrank statistical analyses (GraphPad Prism 4.02; GraphPad Software Inc., San Diego, California, USA). Repeated measurements of Echo data parameters were analyzed using Linear Mixed-Effects model. Each response variable was analyzed for main effects of group and time as well as their interaction. If the group–time interactions were statistically different among groups, comparisons at different time-points were conducted and their outcome was Bonferroni corrected for multiple comparisons. Group differences in hemodynamic indices and histological data among groups were assessed by one-way ANOVA with Bonferroni post-hoc corrections as appropriate. Statistical significance was assumed at *p* < 0.05.

## Results

### Early mortality after coronary ligation and treatment assignment

Among 110 rats subjected to a coronary ligation, 35 animals died within the first 24 h after surgery, and 2 additional rats died within the first 2 weeks after surgery. There was no mortality among the 10 sham-operated rats. Two weeks after surgery for coronary ligation, the remaining 73 rats with MI and 10 rats with sham operation underwent echocardiography, at which time their pre-treatment baseline MI size, LV volumes, and EF were determined. Among these rats, 27 rats, in which MI size was less than 20%, or more than 50% or non-transmyocardial, were excluded from study. The remaining 46 rats with MI were assigned to 2 experimental groups, *n* = 23 each.

### Pre-treatment baseline values

Table 1 lists the Echo-derived pre-treatment values of EDV, ESV, EF, and MI size for each of 3 experimental groups 2 weeks after surgery. The mean value and distribution of MI size, EDV, ESV, and EF data were similar in Control and R, and consisted of significant increases on EDV by 56% and ESV by 187% and a 55% decline in EF compared with Sham.

### Body mass and heart rate

The body mass was similar among 3 groups at all the time-points. The daily average resveratrol consumption was 5 ± 1 mg/kg for whole observation period and the resveratrol diet did not affect the rate of body growth. Heart rate was not different among groups.

### MI expansion

**Fig. 1.**
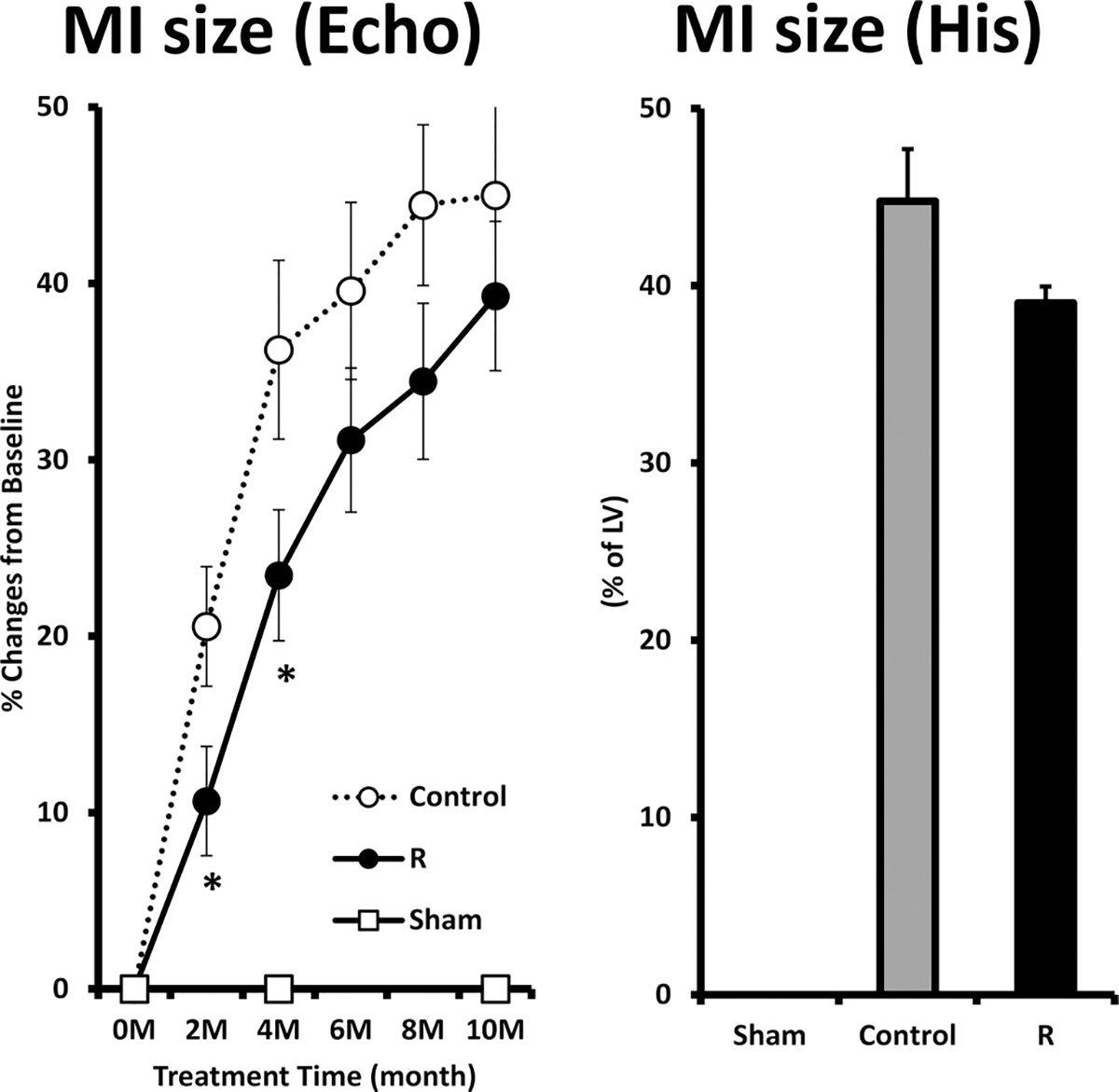
The percent changes of myocardial infarct (MI) size, measured by echocardiography (Echo), from pre-treatment baseline (left panel) and the MI size measured from histological sections (His) at the end of 10-month observation period (right panel). Sham, sham operated; Control, MI rats with regular diet; R, MI rats with resveratrol diet. (*p < 0.05 R vs. Control).

Figure 1, left panel, illustrates the relative change of MI size, assessed by Echo, from pre-treatment baseline during 10-month observation period in Control and R. MI size significantly expanded from 27.6% ± 1.0% to 38.2% ± 1.5% in Control (45% increase) and from 29.9% ± 1.2% to 36.5% ± 0.8% in R (39% increase). The changes in MI size between Control and R were statistically significant at 2 and 4 months of treatment. Figure 1, right panel, shows the MI size, assessed from histological sections at the end of the study, was 45% ± 3% in Control and 39% ± 1% in R (*p* = 0.092), indicating a trend for a significant reduction in MI size in R compared with Control. MI size data derived from Echo and histological measurements were highly correlated (*r* = 0.78).

### LV remodeling and functional deterioration

**Fig. 2.**
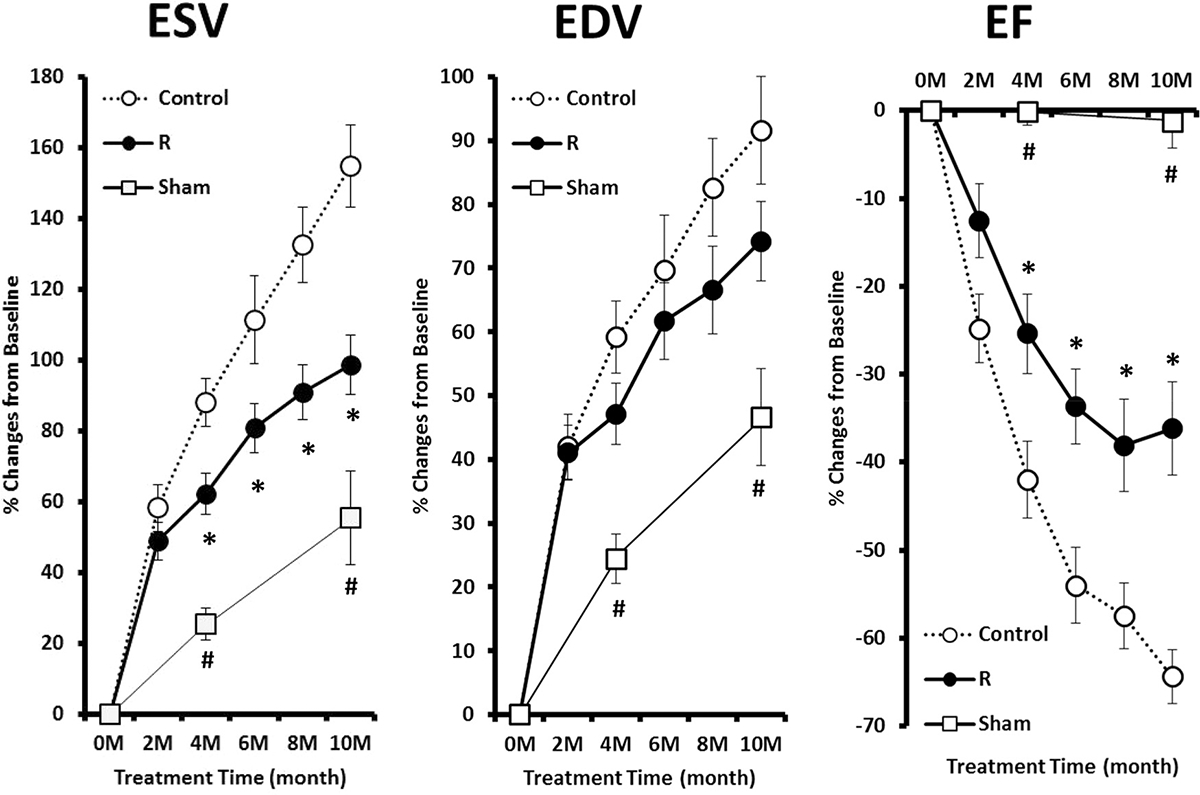
The percent changes of EDV, ESV, and EF from pre-treatment baseline during the 10-month observation period. Sham, sham operated; Control, myocardial infarct (MI) rats with regular diet; R, MI rats with resveratrol diet. (^#^*p* < 0.05 Sham vs. Control and R; *p < 0.05 R vs. Control).

Figure 2 illustrates the relative changes of EDV, ESV, and EF from their respective pre-treatment baseline values during the 10-month observation period in Sham, Control, and R. All the parameters in Sham were significantly different at all time-points compared with those in Control and R. The differences of ESV and EF between R and Control became significant at 4 months of treatment.

During the 10-month observation period, EDV significantly increased from 646 ± 17 μL to 1202 ± 68 μL (*p* < 0.05) in Control, and from 631 ± 18 μL to 1049 ± 28 μL (*p* < 0.05) in R. The relative increases in EDV from pre-treatment baseline were not significantly different between R and Control. ESV significantly increased from 455 ± 17 μL to 1070 ± 61 μL (*p* < 0.05) in Control, and from 469 ± 19 μL to 886 ± 34 μL (*p* < 0.05) in R. The relative increases in ESV from pre-treatment baseline became significantly different between R and Control after 4 months of treatment. EF significantly reduced from 30% ± 1% to 11% ± 1% (*p* < 0.05) in Control, and from 26% ± 1% to 16% ± 1% (*p* < 0.05) in R. The relative reduction in EF from pre-treatment baseline became significantly different between R and Control after 4 months of treatment.

### Hemodynamics and cardiac function

**Fig. 3.**
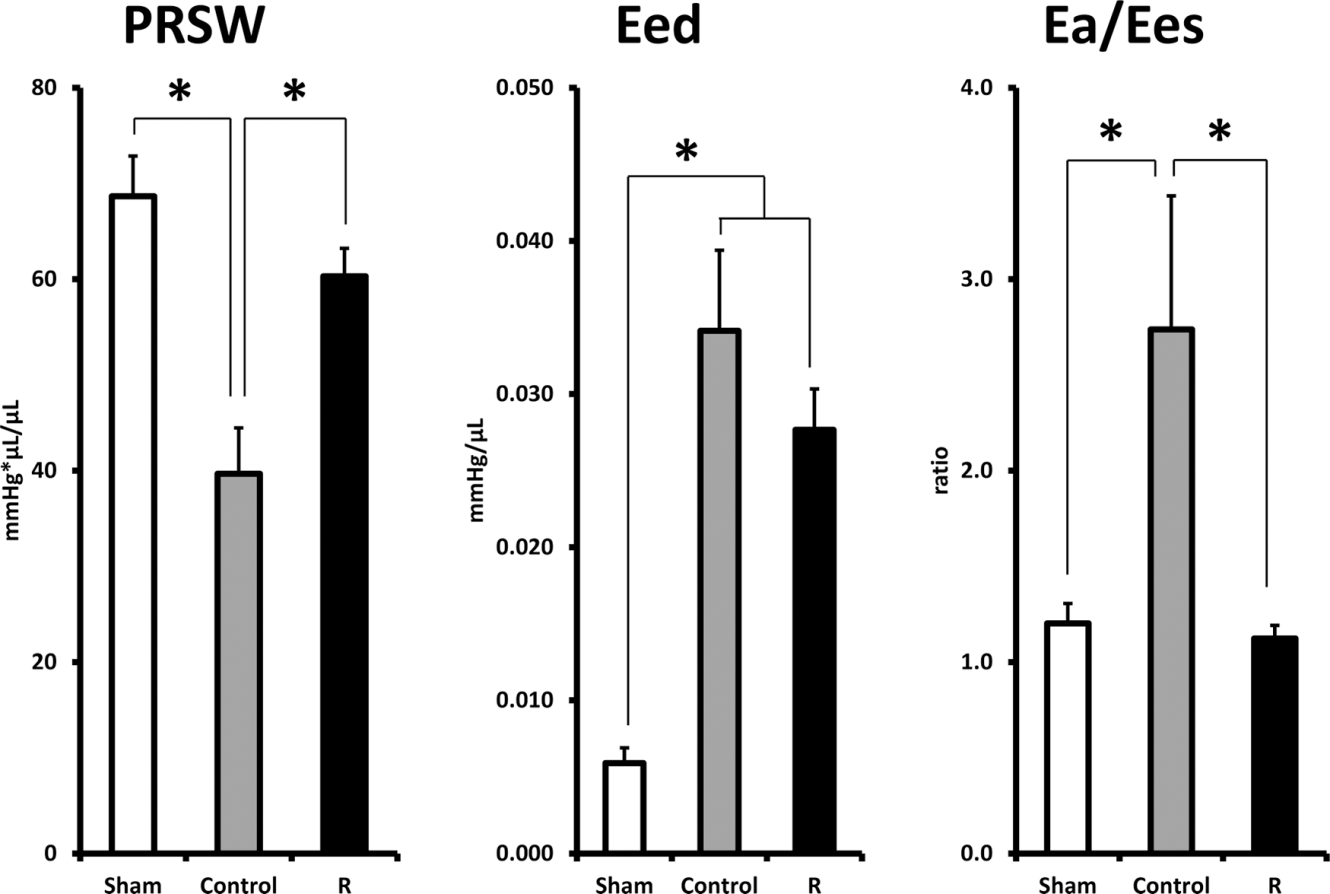
Selected cardiac load-independent indices, PRSW, Eed, and AV-coupling at the end of 10-month observation period. Sham, sham operated; Control, myocardial infarct (MI) rats with regular diet; R, MI rats with resveratrol diet. (*p < 0.05).

Table 2 lists the LV pressure-volume loop parameters prior to sacrifice after 10-month of treatment. Figure 3 shows the PRSW, Eed, and AV-coupling. A comparison of hemodynamic indices of Sham and Control clearly indicates an advanced stage of CHF in Control: a 42% reduction in PRSW indicated a pronounced systolic LV pump dysfunction; a 4.7-fold elevation in Eed reflected an increased diastolic LV stiffness; and a 2-fold increase in AV-coupling reflected a severe inefficiency in transfer of energy from the heart to the arterial tree. In R, both PRSW and AV-coupling returned to their respective levels in Sham and were significantly different compared to that of Control. Eed did not differ between R and Control.

### Myocardial hypertrophy, morphology, myocyte density and size, collagen content

At the end of 10-month of observation period, HM/BM ratio, myocyte density, myocyte diameter, and collagen fraction of the LV posterior wall were significantly different between Sham and Control, indicating a significant remodeling of LV myocardial structure. None of these parameters differed between Control and R (Table 2).

### Aortic stiffness and morphology

**Fig. 4.**
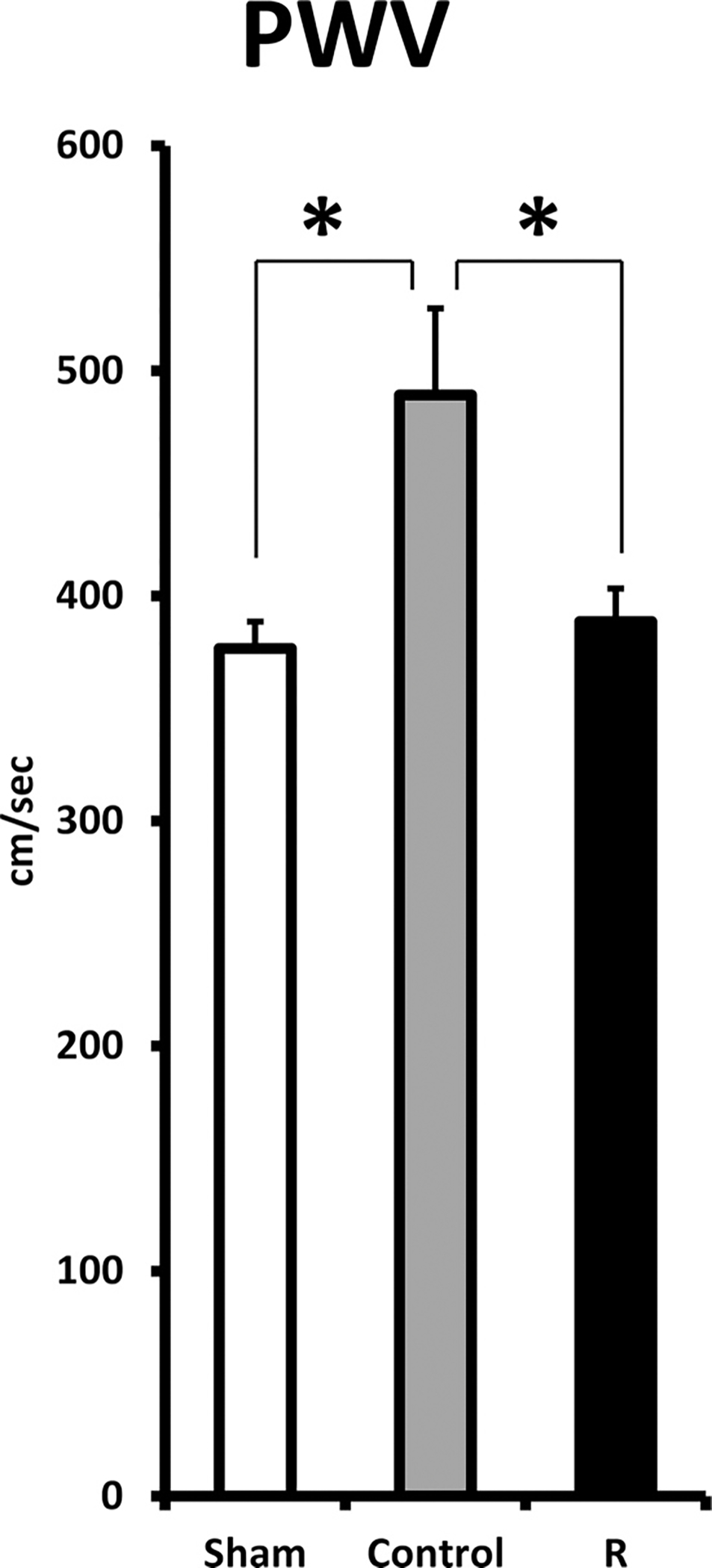
PWV at the end of the 10-month observation period. Sham, sham operated; Control, myocardial infarct (MI) rats with regular diet; R, MI rats with resveratrol diet. (*p < 0.05).

Figure 4 shows the PWV at the end of 10-month observation period prior to sacrifice. PWV was significantly lower in Sham (377 ± 12 cm/s; *p* < 0.05) compared with Control (489 ± 38 cm/s), indicating a significant stiffening of arterial tree in the setting of heart failure. In R, it was 389 ± 15 cm/s, i.e., at the level of Sham, and significantly different compared with Control (*p* < 0.05). The collagen content of thoracic aorta, measured as a fractional area of tunica media, was not significantly different among 3 groups. The lumen diameter and intima-media wall thickness of thoracic aorta did not differ between R and Control (Table 2).

### Mortality

**Fig. 5.**
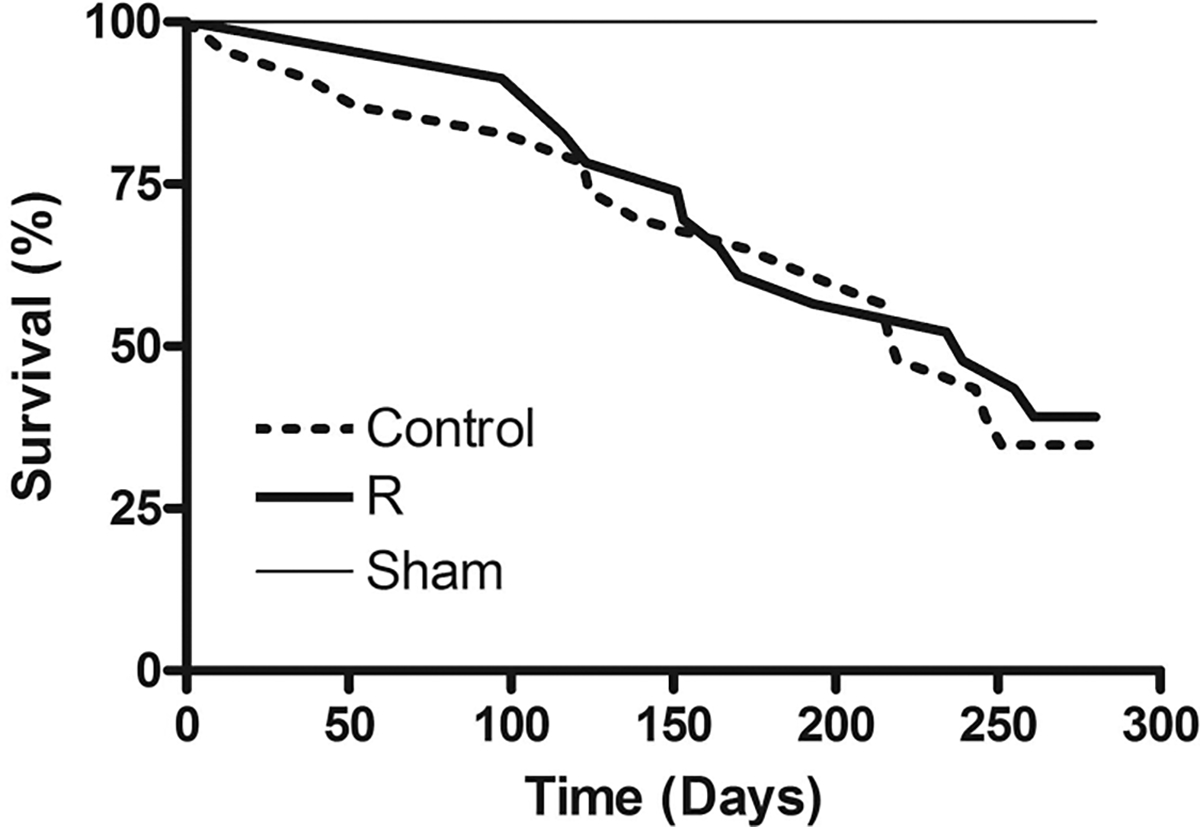
Kaplan–Meier survival curves during the 10-month observation period. Sham, sham operated; Control, myocardial infarct (MI) rats with regular diet; R, MI rats with resveratrol diet. There is no statistical difference between R and Control.

Figure 5 illustrates the Kaplan–Meier survival curves for the Sham, Control, and R during 10-month observation period. None out of 10 rats in Sham, 15 out of 23 rats in Control, and 14 out of 23 rats in R died. There was no statistical difference between Control and R in survival rate during this period. The mortality rate was 65% for Control and 61% for R (*p* = 0.734). The median survival time was 219 days for Control and 239 days for R.

## Discussion

Resveratrol, a red wine extract, had been studied extensively in various diseases and aging models during the last decade (Guerrero et al. 2009). It also had been studied thoroughly in all types of cardiovascular disease models, i.e., ischemia–reperfusion injury, myocardial infarction, hypertrophy, arrhythmias, and heart failure. These studies showed that resveratrol, either given before or after manifestation of disease, could prevent or attenuate the disease progress (Chen et al. 2008; Chong et al. 2015; Hung et al. 2002; Shen et al. 2006; Kanamori et al. 2013; Lin et al. 2008; Liu et al 2005; Robich et al. 2010b; Wojciechowski et al 2010; Xuan et al. 2012). Those remarkable effects were achieved at dosages of resveratrol that many times higher than the amount possibly obtained through daily consumption of resveratrol-rich diet.

Our results showed for the first time that resveratrol, even at a low dosage as a dietary supplement, when used for long term, was beneficial to cardiac structural remodeling and functional decline that accompany chronic heart failure. In rats, following permanent coronary artery ligation, repeated echocardiography showed that long-term resveratrol dietary supplementation significantly reduced LV functional deterioration through increasing LV contractility which was evident from the reduction in the LV end-systolic volume (ESV). Invasive measurements at the end of 10-month of treatment also showed a significantly improved LV systolic function (PRSW) and energy transfer efficacy between heart and arterial tree (AV-coupling).

Also, chronic, low dose resveratrol prevented the elevation of PWV, which is an indication of the stiffening of arterial tree that manifests following CHF. PWV is not only an important index for arterial stiffness but also considered an independent predictor for future cardiovascular events. Beneficial effects of resveratrol in vascular system, i.e, improvement of endothelial function, have been reported in various in vitro and in vivo animal models, but at relatively high dosages (Azorín-Ortuño et al. 2012; Csiszar et al. 2012; Schmitt et al. 2010; Ungvari et al. 2010; Thandapilly et al. 2013). Resveratrol dietary supplement prevented central arterial wall stiffening and inflammation that occurred in non-human primates in response to a metabolic stress induced by a chronic diet high in fat and sucrose (Mattison et al. 2014). Our results showed that low dose of resveratrol in dietary supplement, was still beneficial to vascular remodeling when used for long term. Lack of effect on aortic collagen composition in our study suggests that resveratrol might attenuate the vascular remodeling through improvements of endothelial function or vascular smooth muscle cell stiffening. The beneficial effect of resveratrol in arterial tree might be one of the reasons for the improvements in cardiac systolic function and better energy transfer efficacy while there were lack of significant effect on accumulated mortality, MI expansion, and LV diastolic functional parameters. Nevertheless, the exact mechanism for those beneficial effects of resveratrol is still elusive and out of scope of this study.

Our results are not contradictory to previously published studies that targeting heart failure with lower dosages of resveratrol (Burstein et al. 2007; Magyar et al. 2012; Robich et al. 2010a). These studies showed no improvements in cardiac functional parameters after resveratrol treatment for up to 3 months. Based on the fact that we started to observe significant improvements in cardiac functional parameters only after more than 4 months of treatment, with a slightly prolonged exposure time, these studies could have different outcomes. Our results indicated that it needs a longer exposure time for dietary level of resveratrol to exert its full beneficial effect.

The potency of resveratrol against cardiovascular remodeling in our study is indeed less dramatic than those reported in studies at high therapeutic dosages. Although toxicity of resveratrol has not been reported for high therapeutic dosages, a lower dosage which can mimic a natural occurrence of resveratrol in plants and an amount can be obtained from daily consumption might be a safer choice for long-term usage. Thus, our study results indicated that long-term low dose resveratrol dietary supplement might be a safe, inexpensive addition to current standard therapy for CHF in the clinical setting. Therefore, further studies to test whether resveratrol supplement provides any additional benefit to standard therapy in heart failure patients seems to be warranted.

## Figures and Tables

**Table 1.** Pretreatment baseline values of Echo parameters (2 weeks after coronary ligation).

	Sham (*n* = 10)	Control (*n* = 23)	R (*n* = 23)

EDV (uL)	414±17	646±17*	631±18*
ESV (uL)	165±9	455±17*	469±19*
EF (%)	60±2	30±1*	26±1*
MI size (% of LV)	N/A	28±1	30±1

**Note:** Mean ± SEM.

* *p* < 0.05 vs. Sham.

ESV, end-systolic volume; EDV, end-diastolic volume; EF, ejection fraction; MI size, myocardial infarct size. Sham, sham operated; Control, MI rats with normal diet; R, MI rats with resveratrol enriched diet.

**Table 2.** Hemodynamic and histological indices after 10 months of treatment.

	Sham (*n* = 10)	Control (*n* = 8)	R (*n* = 9)

BM (g)	672±21	676±12	682±11
Hemodynamics			
HR (beats/min)	282±8	317±28	269±14
ESV (μL)	270±20	1121±143*	910±46*
EDV (μL)	556±20	1184±137*	1049±42*
ESP (mmHg)	93±4	86±5	89±4
EDP (mmHg)	4.2±0.5	6.4±1.6	4.0±0.3
SV (μL)	329±19	120±22*	139±10*
EF (%)	59±2	10±1*	14±1*
CO (mL/min)	94±7	39±8*	37±3*
Ea (mmHg/μL)	0.29±0.03	0.77±0.09*	0.69±0.05*
*+dP/dt* (mmHg/sec)	6809±283	4369±332*	5381±299*
*−dP/dt* (mmHg/sec)	−7354±645	−4265±427*	−4841±334*
Tau (ms)	9.1±0.5	11.7±1.1*	10.9±0.5
PRSW (mmHg*μL/μL)	69±4	40±5*	60±3#
Ees (mmHg/μL)	0.27±0.03	0.40±0.07	0.63±0.05*#
Eed (mmHg/μL)	0.006±0.001	0.034±0.005*	0.028±0.003*
Ea/Ees	1.2±0.1	2.7±0.7*	1.1±0.1#
Cardiac histology			
MI size (% of LV)	N/A	45±3	39±1
HM/BM (g/kg)	3.0±0.1	4.1±0.2*	3.8±0.1*
Myocyte density in LVPW (mm^−2^)	319±12	178±7*	202±11*
Myocyte diameter of LVPW(μm)	21±0	37±1*	34±1*
Collagen fraction of LVPW (%)	1.9±0.2	4.1±0.5*	3.4±0.3*
Thoracic aorta histology			
Lumen diameter (μm)	3342±42	3523±61*	3451±19
Medium thickness (μm)	266±9	250±2	248±5
Collagen fraction of medium (%)	11.2±1.0	9.1±0.7	9.2±0.6

**Note:** Data are mean ± SEM

* *p* < 0.05 vs. Sham

# *p* < 0.05 vs. Control.

Sham, sham operated; Control, myocardial infarct (MI) rats with normal diet; R, MI rats with resveratrol enriched diet. BM, body mass; HR, heart rate; ESV, end-systolic volume; EDV, end-diastolic volume; SV, stroke volume; EF, ejection fraction; CO, cardiac output; Ea, arterial elastance; tau, isovolumic relaxation time; PRSW, preload recruitable stroke work; Ees, systolic elastance; Eed, diastolic stiffness; HM/BM, heart mass to body mass ration; LVPW, LV posterior wall
